# Supplementary material for: Exploring the Underlying Mechanism of Ren-Shen-Bai-Du Powder for Treating Inflammatory Bowel Disease Based on Network Pharmacology and Molecular Docking
Source: Pharmaceuticals (Basel). 2022 Aug 23;15(9):1038. doi: 10.3390/ph15091038 (PMC9504917; doi:10.3390/ph15091038)
Supplement: Supplementary file 1 [file pharmaceuticals-15-01038-s001.zip › pharmaceuticals-1842009-supplementary.pdf]

Table S1. Sequences of primers used for qRT-PCR.

Gene		Sequences(5' to 3')
Claudin-1	Forward primer	GCTGGGTTTCATCCTGGCTTCT
	Reverse primer	CCTGAGCGGTCACGATGTTGTC
ZO1	Forward primer	GCCGCTAAGAGCACAGCAA
	Reverse primer	GCCCTCCTTTTAACACATCAGA
Occludin	Forward primer	CTTTGGCTACGGAGGTGGCTAT
	Reverse primer	CTTTGGCTGCTCTTGGGTCTG
CYP1B1	Forward primer	CCACCAGCCTTAGTGCAGAC
	Reverse primer	GGCCAGGACGGAGAAGAGT
PTPN11	Forward primer	AGAGGGAAGAGCAAATGTGTCA
	Reverse primer	CTGTGTTTCCTTGTCCGACCT
CA7	Forward primer	CGCCCTCTACATGGTTCG
	Reverse primer	GGTCAGGGAGCCAGGATA

1) The 10 μL reaction mixtures contain 5 ul ChamQ SYBR qPCR Master Mix (Vazyme, China), 0.2 μl each primer (Tsingke (Wuhan) Co., Ltd, China), 4.1 ul ddH2O, and 0.5 ul cDNA template.
2) The thermocycling steps of qPCR: (1) 95 °C, 3 min; (2) 95 °C, 5 s; (3) annealing temperature, 30 s; (4) 72 °C, 30 s; (5) plate read; (6) repeat steps (2) to (5) 40 more times; (7) melt-curve analysis, 58 °C − 95 °C, 0.5 °C read.
